# Increased lncRNA AFAP1‐AS1 expression predicts poor prognosis in gastric cancer: Evidence from published studies and followed up verification

**DOI:** 10.1002/cam4.5287

**Published:** 2022-09-26

**Authors:** Fujiao Duan, Yilin Li, Yajing Feng, Guanghui Niu, Junhui Chai, Kaijuan Wang

**Affiliations:** ^1^ Department of Medical Research Office and General Surgery Affiliated Cancer Hospital of Zhengzhou University Zhengzhou Henan China; ^2^ Key Laboratory of Tumor Epidemiology of Henan Province Zhengzhou Henan China; ^3^ Department of Nosocomial Infection Management the First Affiliated Hospital of Zhengzhou University Zhengzhou Henan China; ^4^ College of Public Health Zhengzhou University Zhengzhou Henan China

**Keywords:** AFAP1‐AS1, gastric cancer, lncRNA, meta‐analysis, prognosis

## Abstract

**Aim:**

The purpose of this study was to clarify the influence of long non‐coding RNA actin fiber‐associated protein‐1 antisense RNA 1 (lncRNA AFAP1‐AS1) on the prognosis of gastric cancer (GC).

**Methods:**

Based on meta‐analysis, the association between the expression of AFAP1‐AS1 and the prognosis of GC was estimated. GC tissue and non‐cancer tissues from 136 patients were determined by quantitative real‐time reverse transcription polymerase chain reaction (qRT‐PCR) and verified by Gene Expression Profiling Interactive Analysis (GEPIA). Kaplan–Meier and Cox proportional hazards models were conducted to analyze the correlation between AFAP1‐AS1 expression and GC prognosis.

**Results:**

The pooled analysis from five studies revealed that the AFAP1‐AS1 expression was significantly associated with GC overall survival (hazard ratio (HR) = 2.49 and 95% confidence interval (95% CI): 2.02–3.08, *p * <  0.001). Compared with non‐cancer tissues, AFAP1‐AS1 expression level of GC tissues were significantly upregulated (*p * <  0.001), which was confirmed by the results of GEPIA. The area under the receiver‐operating characteristic (ROC) curve was 0.893, and the high expression of AFAP1‐AS1 was correlated with poor prognosis in patients with GC (p = 0.005). Clinical grade (HR = 1.912, 95% CI: 1.246–2.934, p = 0.003), pathologic tumor node metastasis (pTNM) (HR = 2.393, 95% CI: 1.431–4.033, p = 0.001), log odds of positive lymph nodes (LODDS) (HR = 2.910, 95% CI: 1.787–4.793, *p * <  0.001) and AFAP1‐AS1 expression (HR = 2.393, 95% CI: 1.869–3.064, *p * <   0.001) were independent prognostic factors for GC revealed by multivariate Cox‐regression analysis.

**Conclusion:**

This study demonstrated that the AFAP1‐AS1 may be a novel biomarker for the diagnosis and prognosis of GC.

## Graphical abstract

Based on meta‐analysis and TSA, the association between the expression of AFAP1‐AS1 and the prognosis of gastric cancer was estimated. GC tissue and non‐cancer tissues were determined by qRT‐PCR and verified GEPIA. Kaplan–Meier and Cox proportional hazards modelswere conducted to analyze the correlation between AFAP1‐AS1 expression and gastric cancer prognosis. The pooled analysisrevealed that the AFAP1‐AS1 expression was significantly associated with gastric cancer OS.Compared with non‐cancer tissues, AFAP1‐AS1 expression level of gastric cancer tissues were significantlyupregulated, which was confirmed by the results of GEPIA. The high expressionof AFAP1‐AS1 was correlated withpoor prognosis in patients with gastric cancer. Clinical grade, pTNM LODDS andAFAP1‐AS1 expression were independent prognosticfactors for gastric cancer revealed by multivariate Cox‐regression analysis. This study demonstrated that the AFAP1‐AS1 may be a novel biomarker for the diagnosis and prognosis of gastriccancer.

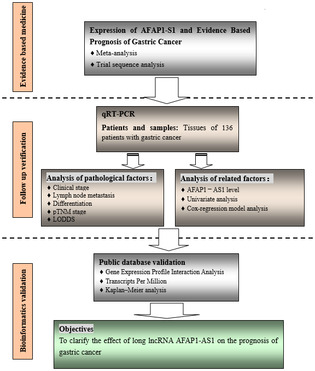

## 1 INTRODUCTION

Gastric cancer (GC) is one of the most common malignant tumors in the digestive system.^1^ GC is the second most common cancer in men and the fourth most common cancer in women. It is also the second leading cause of cancer‐related mortality in China.^2^ Surgery is considered to be an effective radical treatment at present, but due to insufficient markers for early diagnosis, many patients have missed the optimal chance for surgical treatment once diagnosed.^3^ Despite the continuous development and progress of neoadjuvant chemotherapy, targeted therapy and immunotherapy, the therapeutic effect and prognosis are still poor.^4^, ^5^ With the in‐depth study of genetic and molecular biology, more and more long non‐ coding RNAs (lncRNAs) have been excavated in the occurrence, development, and metastasis of cancer, showing extensive potential in cancer diagnosis and treatment.

Although lncRNA has no protein‐coding products, it can play a wide range of regulatory roles, with a length of more than 200 nucleotides.^6^ The imbalance expression of lncRNAs is considered to have a hand in cancer occurrence and progression.^4^, ^7^ It has been proved that lncRNAs can participate in the regulation of genes related to the development of tumor invasiveness through a variety of mechanisms, such as microRNA (miRNA) sponge or competitive endogenous RNA (ceRNA).^8^, ^9^

Studies have confirmed that an lncRNA, actin filament‐associated protein 1‐antisense RNA 1 (AFAP1‐AS1), which was first reported in esophageal cancer plays a role as an oncogene to mediate cell proliferation, migration, and invasion.^10^ In addition, AFAP1‐AS1 has been found to play a key role in various types of human cancers, including lung cancer,^11^ breast cancer,^12^ colorectal cancer^13^ and hepatocellular carcinoma^14^ has also been discussed. However, the expression and prognosis effect of AFAP1‐AS1 in GC remain unclear.

In the published studies, there are few studies focusing on the relationship between AFAP1‐AS1 expression and the prognosis of GC. The specific target of AFAP1‐AS1 and its indicating role in the occurrence and prognosis of GC are still scattered, thus further integrated study is needed. In the present study, we conducted a quantitative systematic evaluation, and then evaluated the effect of AFAP1‐AS1 on the clinicopathological features and prognosis of GC by using the follow‐up population, and further confirmed the results of quantitative evaluation.

## 2 MATERIALS AND METHODS

This study was performed based on the Meta‐analysis of Observational Studies in Epidemiology (MOOSE).^15^ In order to clarify the clinical significance of AFAP1‐AS1 in GC, we completed the study design according to the principles of population, intervention, comparison, results and study design (PICO).

### 2.1 Meta‐analysis of AFAP1‐AS1

To obtain the correlation between AFAP1‐AS1expression and the prognosis of GC, a systematic literature searching was implemented using PubMed, EMBASE, Cochrane Library, science network, CNKI (Chinese), Wanfang (Chinese), VIP (Chinese) and CBM (Chinese).

The search included the following three groups of terms: “tumor” and “cancer” and “carcinoma”; “gastric cancer”, “gastric tumor” and “gastric carcinoma” or “stomach cancer”, “stomach tumor” and “stomach carcinoma”; “long noncoding RNA AFAP1‐AS1” and “lncRNA AFAP1‐AS1”; “clinical outcome”, “survival” and “prognosis”. Searches were conducted using all combinations of at least one term from each group.

The included studies met the following criteria: (1) Cohort studies that investigated associations between AFAP1‐AS1 expression and GC with OS, DFS and/or PFS, (2) GC were divided into two groups according to the high and low expression of AFAP1‐AS1, (3) hazard ratios (HRs) and 95% confidence intervals (95% CIs) were reported or could be calculated from given data, (4) published in English or Chinese. If the data overlap with other published literatures, we select a newly published and/or larger sample article.

For the OS, the starting point was diagnostic time, and operation day or treatment time for others. When HR and/or 95% CI were not reported, the methods of Parmar at al.^16^ and Tierney et al.^17^ were used for extrapolation.

### 2.2 Patients and samples

GC and corresponding adjacent tissues of 136 patients with GC from the First Affiliated Hospital of Zhengzhou University and the Affiliated Cancer Hospital of Zhengzhou University from April 2015 to July 2016 were selected. All patients did not receive received radiotherapy and chemotherapy before surgical treatment. The specimens were stored in a −80°C refrigerator immediately after in vitro. This study was approved by medical ethics committee of Zhengzhou University. We obtained written informed consent from patients before taking samples.

The log odds of positive lymph nodes (LODDS) stage^18^ based on the number of positive lymph nodes (pnod) and total number of lymph node examinations(tnod).

$$\text{LODDS}=\log\left[\left(\text{pnod}+0.5\right)/\left(\text{tnod}-\text{pnod}+0.5\right)\right].$$

This study applies the staging standard formulated by Sun et al.^18^ LODDS1(≤ −1.5), LODDS2 (−1.5 < LODDS≤−1.0), LODDS3 (−1.0 < LODDS ≤ −0.5), LODDS4 (−0.5 < LODDS ≤ 0), LODDS5 (>0).

### 2.3 Quantitative real‐time reverse transcription PCR

Total RNA was extracted from cancer tissues and adjacent tissues with Trizol reagent (Invitrogen), and cDNA was reverse transcribed with Prime‐Script RT kit (Takara). Then, the cDNA was used as a template for RNA reverse transcription. Quantitative real‐time reverse transcription PCR (qRT‐PCR) was performed by SYBR Green dye method. qRT‐PCR was performed by three‐step method. The reaction conditions were as follows: Pre denaturation at 95°C for 10 min, continuous denaturation for 10 s, annealing at 57°C for 20 s, and extension at 72°C for 15 s. Set 3 parallel samples for each sample to be tested and calculate the average value. Primer 5.0 was used to design primers. AFAP1‐AS1 forward was 5′‐CCGTCCATGCGGAAGATC‐3′, and the reverse was 5′‐ATGGCCAGCGGGAAGAC‐3′. The forward of GAPDH was 5′‐AGCCACATCGCTCAGACAC‐3′, and the reverse was 5′‐GCCCAATACGACCAAATCC‐3′. The qRT PCR reaction was performed with ABI 7500 system and SYBR Green PCR master mix (Applied Biosystems). The multiple ratio of AFAP1‐AS1expression in GC and corresponding adjacent tissues was analyzed by 2^−ΔΔCt^.

### 2.4 Statistical analysis

Revman 5.3.5 (Cochrane Collaboration), Statistical Package for the IBM SPSS Statistics 23.0, STATA 13.1MP (StataCorp LP) and GraphPad Prism 8.0 (GraphPad Software, Inc.) software were used to conduct all statistical analyses. Inter‐study heterogeneity was quantified using Q‐tests and the I‐squared (*I* ^2^) statistic. According to the results of heterogeneity analysis, a fix effects or random model was conducted. In the absence of significant heterogeneity (*P*_heterogeneity_ ≥ 0.10 or *I* ^2^ ≤ 50%), a fixed‐effects model was used to assess the combined effect size (HR), otherwise (*P*_heterogeneity_ < 0.1 and *I* ^2^ > 50%) the random‐effects model was performed. Begg's and Egger's tests were used to evaluate the publication bias. The sample size required (required information size, RIS) for meta‐analysis was calculated by applying trial sequence analysis (TSA) (version 0.9.5.10 Beta).

Continuous data were presented as mean ± SD, and compared using paired Student's *t*‐test. The relationship between AFAP1‐AS1 expression and prognostic factors was performed by Person's chi‐square (*χ* ^2^) test or Fisher's exact test. Receiver operating characteristic (ROC) curve analysis was used to evaluate the effect of AFAP1‐AS1 on different cancer tissues and adjacent tissues.

The survival curve was estimated by Kaplan–Meier (KM) method, and log‐rank test was applied for inter group comparison. Binary unconditional logistic regression was used to detect the relationship between AFAP1‐AS1 expression and a single prognostic factor. Cox proportional hazards regression model was used to calculate HRs and 95% CIs, and adjusted for clinicopathological prognostic factors.

All *p*‐values were two‐sided and *p* < 0.05 was considered statistically significant.

## 3 RESULTS

### 3.1 Meta‐analysis

A total of 177 records were retrieved according to the literature search strategy. After systematic screening and identification of processes, five studies^19^, ^20^, ^21^, ^22^ (included the present study) were finally confirmed and pooled analysis. The quality assessment of qualified studies was evaluated based on Newcastle‐Ottawa Scale (NOS) (Table S1) and Quality In Prognosis Studies (QUIPS) (Table S2). All included studies were from 2017 to 2021. The detection methods of AFAP1‐AS1 were qRT‐PCR. Frozen tissues were used to assess AFAP1‐AS1 expression in OS (Table 1).

**TABLE 1:** Clinical features of included studies

Author	Year	Country	Number (OS)	Histology	TNM stage	Sample	Assay	Follow‐up (months)	Cut‐off	Outcome	Total score^a^	Level of evidence^b^
Duan et al^c^	2021	China	136	Gastric cancer	I–IV	Frozen tissue	qRT‐PCR	60	Median	HR/SC	7	2b
Ma et al^20^	2020	China	120	Gastric cancer	NA	Frozen tissue	qRT‐PCR	60	Median	SC	5	2b
Zhao et al^21^	2018	China	80	Gastric cancer	I–IV	Frozen tissue	qRT‐PCR	36	Normal	SC	8	1b
Ye et al^22^	2018	China	66	Gastric cancer	I–IV	Frozen tissue	qRT‐PCR	26	Median	HR	7	2b
Feng et al^8^	2017	China	91	Gastric cancer	I–IV	Frozen tissue	qRT‐PCR	66	Normal	HR/SC	7	2b

Abbreviations: DFS, disease free survival; HR, hazard ratio; NA, not available; OS, overall survival; PFS, progressive free survival; qRT‐PCR, quantitative real‐time PCR; SC, survival curve; TNM, tumor node metastasis.

^a^ Quality assessment of included studies according to the Newcastle–Ottawa Scale (Table S1).

^b^ The levels of evidence were estimated for all eligible studies with the Oxford Centre for Evidence‐Based Medicine criteria (Table S2).

^c^ The present case–control study.

**FIGURE 1:**
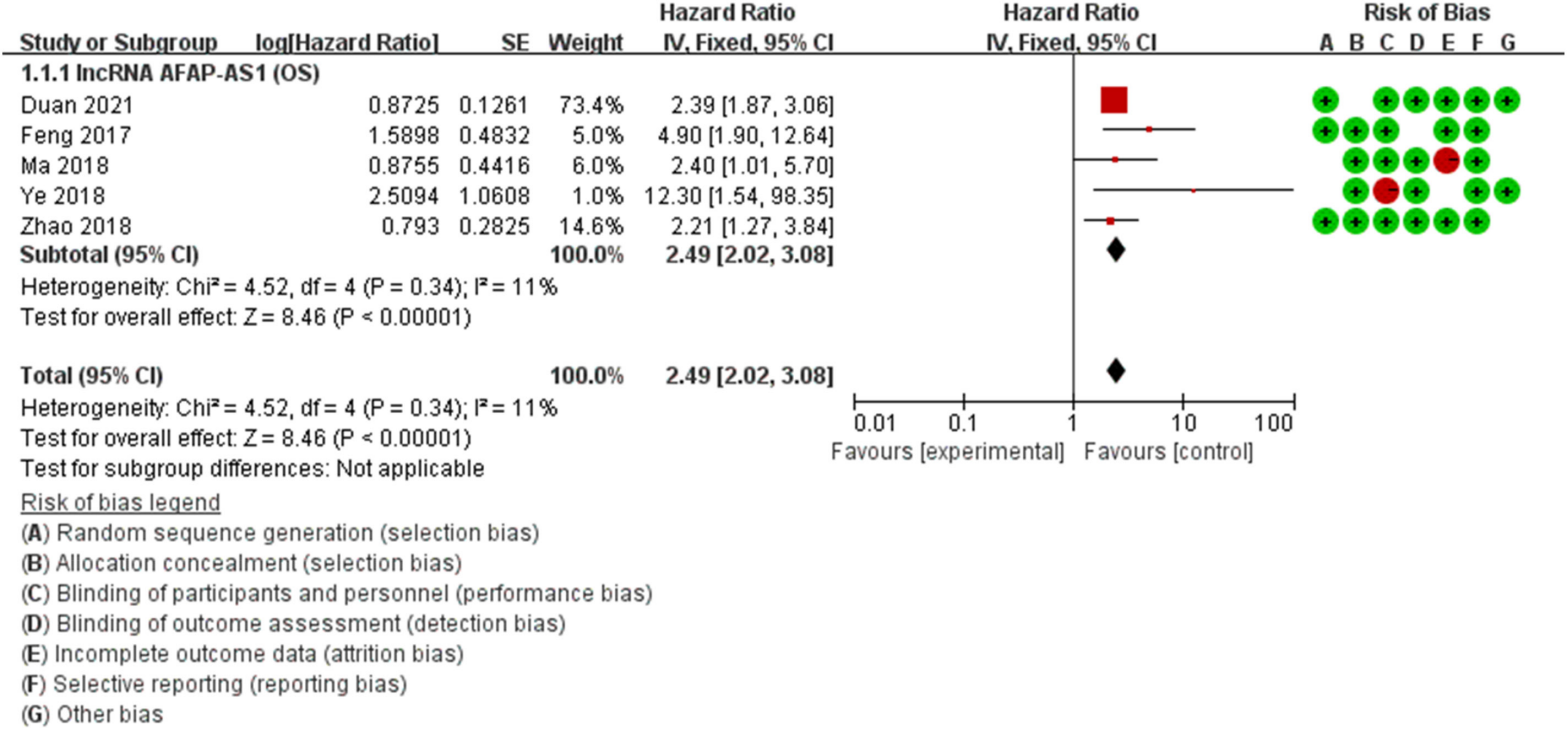
Forest plots for the correlation between AFAP1‐AS1 expression and prognosis of gastric cancer patients.

The OS heterogeneity of AFAP1‐AS1 was not statistically significant (*p*_heterogeneity_ = 0.34 and *I* ^2^ = 11%) (Figure 1). Overall, the combined analysis of the 5 studies revealed that AFAP1‐AS1 expression was significantly associated with OS (HR = 2.49, 95% Cl: 2.02–3.08, *p* < 0.001) (Figure 1).

To assess the stability of the results, we performed a one‐way sensitivity analysis by successively missing each study to clarify the impact of a single data set on the combined HR. The results showed that there was no substantial change in the corresponding combined HRs (Figure S1), which indicated that our results were robust. In order to evaluate the publication bias, we conducted Begg's funnel test (*z* = 2.20, *p* = 0.27), and the results showed no obvious signs of asymmetry, and the results were verified by Egger's test (*t* = 1.89, *p* = 0.155).

TSA was used to estimate the sample size of meta‐analysis, and the result showed that the accumulation curve *Z* crossed the traditional boundary value, but did not cross the TSA boundary value, and its accumulated information did not reach the expected information (RIS, 8276), indicating that the meta‐analysis may have reached a false positive conclusion, and more studies need to be included to confirm the relationship between the expression of AFAP1‐AS1 and the prognosis of GC (Figure S2).

### 3.2 Expression and prognosis of AFAP1‐AS1 in gastric cancer

#### 3.2.1 AFAP1‐AS1 expression and clinicopathological features

The clinicopathological features of patients with GC include age, gender, history of family, tumor size, clinical grade, lymph node metastasis, and histological differentiation were presented in Table 2. The results indicated that there was no relationship between the expression of AFAP1‐AS1 and age (*p* = 0.161), gender (*p* = 0.841), family history (*p* = 0.121) and serosal infiltration (*p* = 0.135) in GC tissues, but related with tumor sizes (*p* = 0.002), clinical stage (*p* = 0.003), lymph node metastasis (*p* = 0.006), differentiation (*p* < 0.001), pathologic tumor node metastasis (pTNM) (*p* = 0.001) and LODDS (*p* < 0.001) (Table 2).

**TABLE 2:** The clinical characterization of patients with gastric cancer

Clinical features	*N* (136)	Expression of AFAP1‐AS1		*χ* ^2^ */t*	*p* ^a^
		High (68)	Low (68)		
Age, years				0.161	1.966
Mean ± SD		61.10 ± 11.29	59.38 ± 1163	0.875	0.383^b^
<60	54	23 (34.80%)	31 (46.56%)		
≥60	82	45 (66.20%)	37 (53.44%)		
Gender				0.040	0.841
Male	103	51 (75.00%)	52 (76.47%)		
Female	33	17 (25.00%)	16 (23.53%)		
Family history				2.401	0.121
Positive	25	16 (23.53%)	9 (13.24%)		
Negative	111	52 (76.47%)	59 (86.76%)		
Tumor sizes (*d* /cm)				9.836	0.002
<5	80	31 (45.78%)	49 (72.06%)		
≥5	56	37 (54.22%)	19 (27.94%)		
Clinical stage				8.819	0.003
I–II	84	29 (42.65%)	55 (80.88%)		
III–IV	52	39 (57.35%)	13 (19.12%)		
Cli				7.696	0.006
Positive	58	37 (54.41%)	21 (30.88%)		
Negative	78	31 (45.59%)	47 (69.12%)		
Differentiation				22.12	<0.001
Well	46	11 (16.18%)	35 (51.47%)		
Moderate	60	34 (50.00%)	26 (38.24%)		
Poor	30	23 (33.82%)	7 (10.29%)		
pTNM stage				6.585	0.010
I–II	44	15 (22.06%)	29 (43.79%)		
III–IV	92	53 (77.94%)	39 (56.21%)		
Serosal infiltration				6.103	0.013
Yes	54	33 (48.53%)	19 (27.94%)		
No	82	35 (51.47%)	49 (72.47%)		
LODDS				10.350	0.033^c^
LODDS1	51	19 (27.94%)	30 (44.12%)		
LODDS2	37	13 (19.12%)	20 (29.41%)		
LODDS3	25	18 (26.47%)	11 (16.18%)		
LODDS4	16	12 (17.65%)	5 (7.35%)		
LODDS5	7	6 (8.82%)	2 (2.94%)		

*Note: p*‐values were two‐sided and *p* < 0.05 was considered statistically significant.

Abbreviations: LODDS, log odds of positive lymph node; pTNM, pathologic tumor node metastasis; SD, standard deviation.

^a^ Person's chi‐square (*χ*)^2^ test.

^b^ 2‐Sample *t*‐test.

^c^ Fisher's exact test.

#### 3.2.2 AFAP1‐AS1 expression in gastric cancer tissue

**FIGURE 2:**
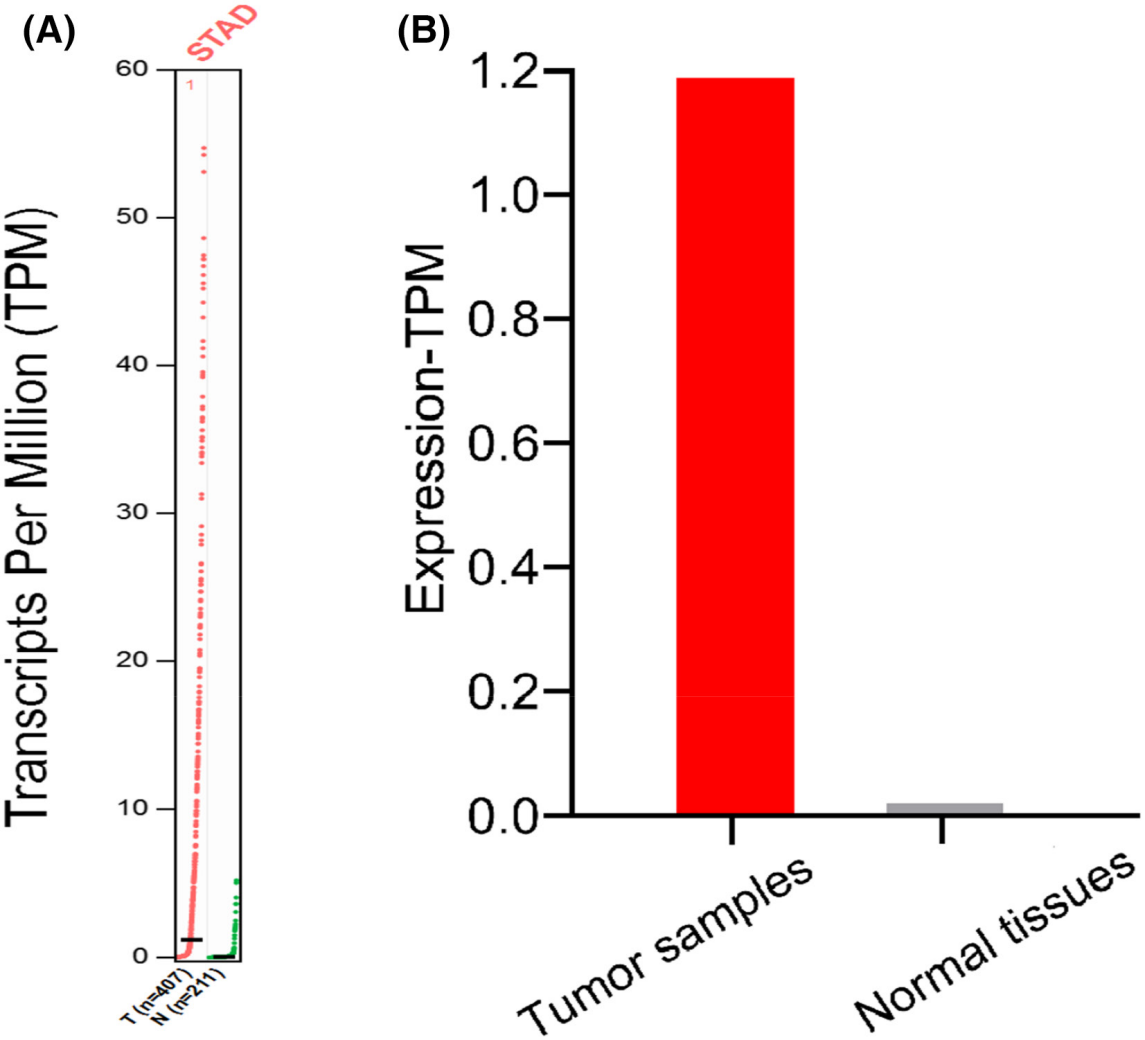
The AFAP1‐AS1 expression and prognosis in gastric cancer. (A) AFAP1‐AS1 expression in gastric cancer tissues (407) and adjacent tissues (211) based on Transcripts Per Million (TPM). (B) Data collected from the GEPIA database presented the relative AFAP1‐AS1 expression in gastric cancer tissues and matched non‐tumorous tissues.

The bioinformatics tool “GEPIA” (Gene Expression Profile Interaction Analysis) was used to analyze 617 cases of GC and normal tissues. The results showed that AFAP1‐ AS1 expression in GC tissues were significantly higher than that in adjacent normal tissues based on Transcripts Per Million (Figure 2A) and Match TCGA normal and GTEx data (Figure 2B).

**FIGURE 3:**
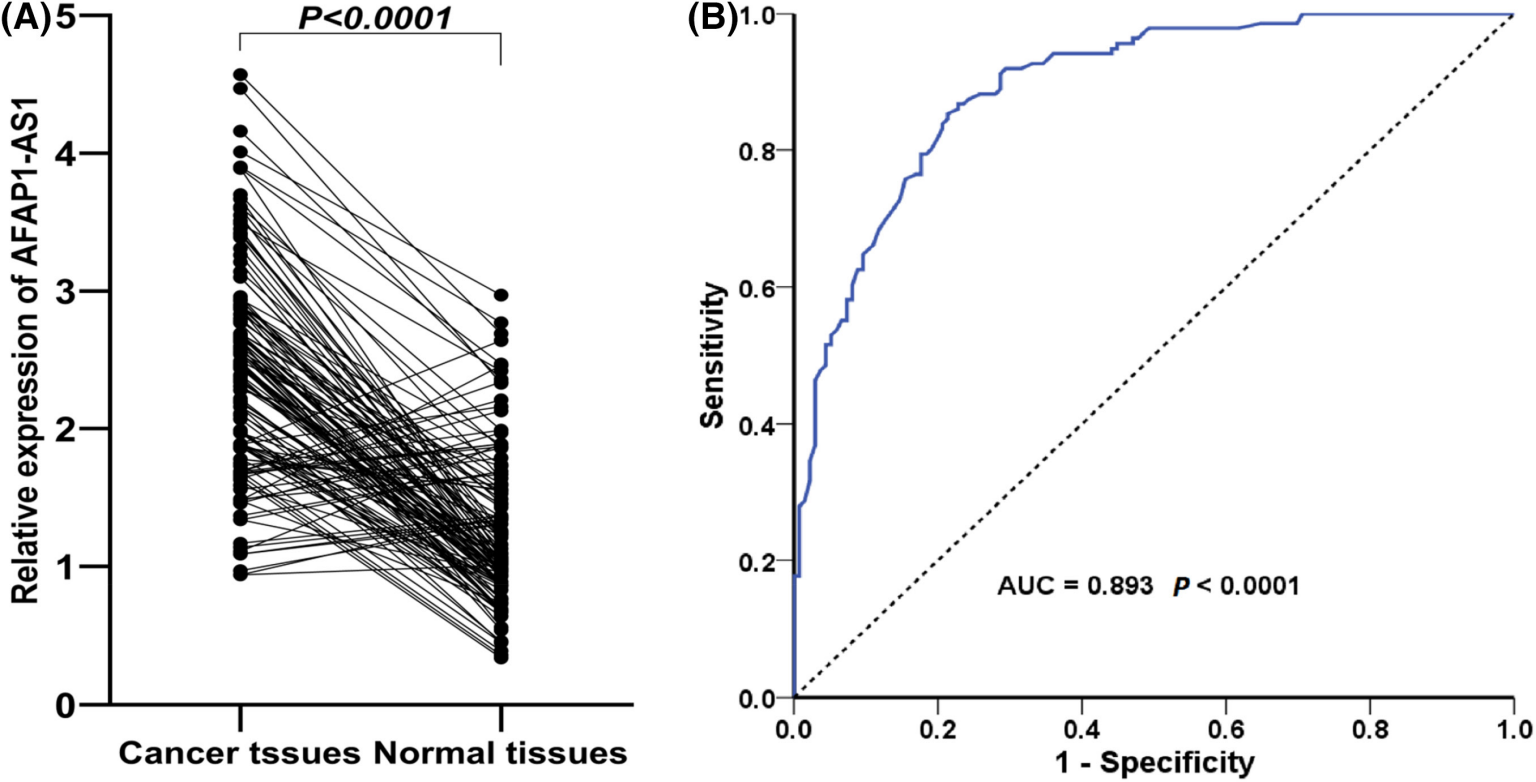
(A) AFAP1‐AS1 expression in 136 pairs of gastric cancer tissues and adjacent tissues. (B) Receiver operating characteristic (ROC) curve indicated that the AFAP1‐AS1 expression has an AUC of 0.893 in distinguishing the gastric cancer tissues from the adjacent tissues.

The qRT‐PCR was applied to detect the AFAP1‐ AS1 expression in 136 patients with GC. The results indicated that AFAP1‐AS1expression was significantly overexpressed in GC tissues compared with adjacent tissues (*p* < 0.001) (Figure 3A). To explore whether the expression characteristics of AFAP1‐AS1 can be used as a biomarker for the detection of GC, we performed ROC curve analysis. The results showed that the AFAP1‐AS1 expression may be used as a diagnostic marker of GC (area under the curve 0.893) (Figure 3B).

#### 3.2.3 AFAP1‐AS1 expression and prognosis in gastric cancer tissue

**FIGURE 4:**
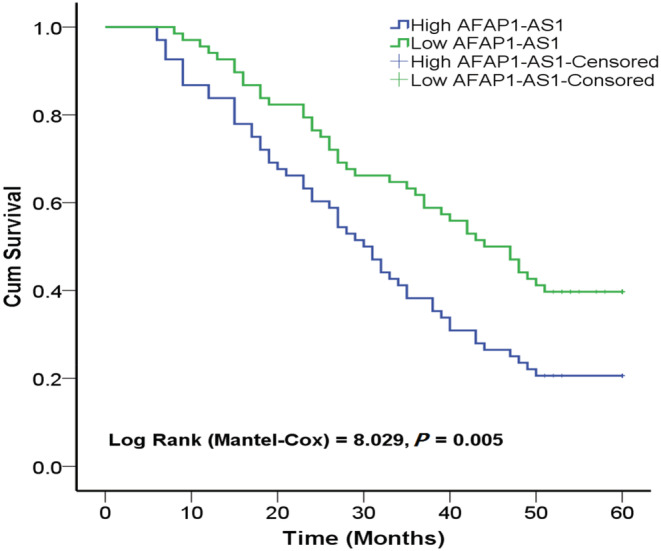
Kaplan–Meier 5‐year overall survival curves based on AFAP1‐AS1 expression.

Log‐rank test (Kaplan–Meier analysis) indicated that the 5‐year OS rate of low AFAP1‐AS1 group was significantly higher than that of high AFAP1‐AS1 group, indicating that patients with high AFAP1‐AS1 expression had poor prognosis (*p* = 0.005, Figure 4).

For univariate analysis, the significant factors (age, family history, clinical stage grade, lymph node metastasis, differentiation, pTNM stage and LODDS) were included in multivariate Cox‐regression analysis. The results suggested that clinical grade (HR = 1.912, 95% CI: 1.246–2.934, *p* = 0.003), pTNM stage (HR = 2.393, 95% CI: 1.431–4.033, *p* = 0.001), LODDS (HR = 2.910, 95% CI: 1.787–4.793, *p* < 0.001) and the AFAP1‐AS1 expression (HR = 2.393, 95% CI: 1.869–3.064, *p* < 0.001) were independent prognostic factors for patients with GC (Table 3).

**TABLE 3:** Univariate and Cox‐regression model analysis of overall survival in gastric cancer patients

Characteristics	Categories	Univariate analysis		Multivariate analysis	
		HR (95% CI)	*p*	HR (95% CI)	*p*
Age	≥60 vs. <60	2.647 (1.118,6.259)	0.027	1.066 (0.691, 1.645)	0.772
Gender	Female vs. male	1.204 (0.479,3.026)	0.693		
Family history	Positive vs. negative	4.246 (1.403, 12.848)	0.010	1.120 (0.652, 1.924)	0.683
Tumor size	≥5 vs. <5	2.020 (0.837, 4.879)	0.118		
Clinical grade	III–IV vs. I–II	3.281 (1.448, 7.433)	0.004	1.912 (1.246, 2.934)	0.003
Lymph node metastasis	Positive vs. negative	4.793 (2.209, 11.326)	<0.001	1.120 (0.652, 1.924)	0.615
Histological differentiation	Poor vs. moderate vs. well	3.720 (1.554, 8.905)	0.003	1.420 (0.901, 2.237)	0.130
pTNM stage	III–IV vs. I–II	4.572 (1.929, 10.841)	<0.001	2.393 (1.431, 4.033)	0.001
Serosal infiltration	Yes vs. no	2.167 (0.786, 5.978)	0.135		
LODDS	LODDS2‐5 vs. LODDS1	5.815 (2.312, 14.623)	<0.001	2.910 (1.787, 4.793)	<0.001
AFAP1‐AS1 level	High vs. low	4.167 (2.385, 7.281)	<0.001	2.393 (1.869, 3.064)	<0.001

*Note*: *p*‐values were two‐sided and *p* < 0.05 was considered statistically significant.

Abbreviations: CI, confidence interval; HR, hazard ratio; LODDS, log odds of positive lymph node; pTNM, pathologic tumor node metastasis.

## 4 DISCUSSION

LncRNA is considered as an emerging participant in promoting the initiation and progression of cancer in recent years. Depending on the specific situation, lncRNA plays different roles in inhibiting or promoting the occurrence and progression of cancer.^23^, ^24^, ^25^ It is shown that the expression imbalance, deletion or mutation of lncRNA is intensively associated with a variety of biological behaviors of GC.^26^ Previous studies have found that the up regulation of AFAP1‐AS1 is related to the poor prognosis of patients with non‐small cell lung cancer.^27^ It is also reported that its up‐regulation is related to the tumor invasion and caused poor prognosis of nasopharyngeal carcinoma.^28^ However, its biological role and prognosis significance in GC are still blurred. This study confirmed the AFAP1‐AS1 expression level of GC and evaluated its correlation with clinical factors.

We evaluate the relationship between the AFAP1‐AS1 expression and the prognosis of GC using quantitative systematic evaluation. The results showed that the AFAP1‐AS1 expression was interrelated with the prognosis of GC. In order to further confirm the combined results of the first step, we used TSA to estimate the RIS for meta‐analysis, and detected the expression level of AFAP1‐AS1 in cancer tissues and adjacent tissues by qRT‐PCR. It was found that AFAP1‐AS1 was relatively highly expressed in GC. The average expression of AFAP1‐AS1 of GC was significantly higher than that of adjacent tissues, ROC analysis indicated that AFAP1‐AS1 expression may be used as a diagnostic marker for GC (area under the curve 0.893), and patients with high AFAP1‐AS1 expression had poor prognosis. This result was further verified by bioinformatics (GEPIA). Multivariate Cox regression analysis confirmed that the clinical stage, pTNM, LODDS, and AFAP1‐AS1 expression were correlated with the GC, and were independent prognostic factors. The results were highly consistent with the founding of our meta‐analysis, suggesting that AFAP1‐AS1 is a potential prognostic marker of GC. This study confirmed the potential value of AFAP1‐AS1 in the early diagnosis and prognosis anticipation and laid a foundation for further exploring the functional mechanism of AFAP1‐AS1 in GC.

Actin filament‐associated protein 1 (AFAP1‐AS1, formerly known as afap‐110) is an antisense lncRNA, an actin cross‐linked protein, and can bind to CSRC. It belongs to AFAP1, AFAP1 class‐1, and AFAP1 like‐2/xb‐130 family members.^29^, ^30^ Wu et al.^10^ first found that AFAP1‐AS1 was overexpressed in esophageal cancer and Barrett's esophagus due to its gene site hypomethylation. After that, Zeng et al.^11^ analyzed five groups of previously published gene expression profiles (GEP) of lung cancer in the high‐throughput microarray expression profile database (GEO). The results showed AFAP1‐AS1 was most significantly expressed in lung cancer, which was related to poor prognosis. A quantitative systematic review was performed by Liu et al.^31^ that included 1017 patients from eight studies. The results revealed that tumor patients with high AFAP1‐AS1 expression had a higher risk of lymph node and distant metastasis. The OS rate, PFS rate, and recurrence free survival (RFS) rate of patients with high AFAP1‐AS1expression were lower than those of patients with low expression, and the high AFAP1‐AS1expression was related with worse clinical prognosis. Therefore, AFAP1‐AS1 may become a potential new biomarker, which could be applied to predict the clinical prognosis of cancer. In addition, Luo et al.^32^ showed that AFAP1‐AS1 could up‐regulate the expression in esophageal cancer, promote the proliferation of cancer cells and inhibit their apoptosis. Nevertheless, there are few studies on AFAP1‐AS1 in GC and lack of prospective studies.

It should be noted that there are still some deficiencies in this study. First, the number of studies included in the meta‐analysis in the first stage of our study is insufficient to perform subgroup analysis, which has a certain impact on the statistical effectiveness of the data. Second, this study involves only one marker, AFAP1‐AS1, which has limited prognostic ability in predicting GC. Future studies should include multiple markers to build a prognostic prediction model, which will be more precise and better for clinical applications. Third, the correlation between the AFAP1‐AS1 expression and the prognosis of GC, as well as the clinical sample data and follow‐up analysis, animal experiments are needed to further verify the experimental results. Finally, the specific molecular mechanism has not been involved in this study. In future studies, it is necessary to clarify the specific mechanism of AFAP1‐AS1 promoting the proliferation of GC.

## 5 CONCLUSION

In summary, the meta‐analysis found that the high expression of AFAP1‐AS1 was related with the prognosis of GC. The level of AFAP1‐AS1 expression in GC tissues was significantly higher than that in non‐cancer tissues. Patients with high AFAP1‐AS1 expression had a poor prognosis in GC. The clinical grade, pTNM, LODDS, and AFAP1‐AS1 were independent prognostic factors for patients with GC. Our founding indicated that AFAP1‐AS1 may be a novel biomarker for the diagnosis and prognosis of GC. In order to explore whether AFAP1‐AS1 could be used as a therapeutic target for GC, mechanism and more preclinical studies should be carried out in future studies.

## AUTHOR CONTRIBUTIONS

Fujiao Duana and Kaijuan Wang were involved in design and conception. Guanghui Niu and Yajing Feng were involved in statistical analyses. Junhui Chai and Yilin Li analyzed and interpreted the data. Fujiao Duana wrote the original draft. All authors revised the manuscript and approved the final manuscript.

## CONFLICT OF INTEREST

The authors declare no conflicts of interest.

## ETHICS APPROVAL AND CONSENT TO PARTICIPATE

The study was approved by the ethics committee of Zhengzhou University (ID: ZZUIRB 2018–0075). This study obtained written informed consent from participants to participate in the study.

## CONSENT FOR PUBLICATION

Written informed consent was obtained from all subjects before enrollment.

## Supporting information


Data S1

## Data Availability

The data sets that were used and/or analyzed during the present study are available from the first or corresponding author.
