# Supplementary material for: Increased lncRNA AFAP1‐AS1 expression predicts poor prognosis in gastric cancer: Evidence from published studies and followed up verification
Source: Cancer Med. 2022 Sep 26;12(4):4227–35. doi: 10.1002/cam4.5287 (PMC9972132; doi:10.1002/cam4.5287)
Supplement: Supplementary file 1 — Data S1 [file CAM4-12-4227-s001.docx]

**List for supplementary materials**

**Supplementary Tables**

**Supplementary Table 1** Quality assessment of included studies based on the Newcastle–Ottawa Scale (NOS) for assessing the quality of cohort studies.

**Supplementary Table 2** Quality assessment of included studies based on the Quality In Prognosis Studies (QUIPS).

**Supplementary Figures**

**Supplementary Figure 1** Sensitivity analysis for overall survival of AFAP1-AS1 expression.

**Supplementary Figure 2** Adjusted boundaries of trial sequence analysis.

Supplementary Table 1 Quality assessment of included studies based on the Newcastle–Ottawa Scale (NOS) for assessing the quality of cohort studies.

| Study | Selection  (score) |  |  |  |  | Comparability  (score) |  | Exposure  (score) |  |  |  |
| --- | --- | --- | --- | --- | --- | --- | --- | --- | --- | --- | --- |
|  | Representativenes  of the exposed cohort | Selection of the  non-exposed cohort | Ascertainment  of exposure | Outcome of interest was not present at  start of study |  | Based on the  design or analysis ^a^ |  | Assessment  of outcome | Follow-up long  enough for  outcomes to occur | Adequacy of  follow-up  of cohorts | Total  Score^b^ |
| Duan et al^a^ | 1 | 0 | 1 | 1 |  | 2 |  | 1 | 1 | 1 | **8** |
| Ma et al[20] | 1 | 0 | 1 | 1 |  | 0 |  | 1 | 1 | 0 | **5** |
| Zhao et al[21] | 1 | 0 | 1 | 1 |  | 0 |  | 1 | 1 | 1 | **8** |
| Ye et al[22] | 1 | 0 | 1 | 1 |  | 2 |  | 1 | 0 | 1 | **7** |
| Feng et al[8] | 1 | 0 | 1 | 1 |  | 2 |  | 1 | 1 | 0 | **7** |

^a^ When there was no statistical significance in the response rate between case and control groups by using a chi-squared test (*P* > 0.05), one point was awarded.

^b^Total score was calculated by adding up the points awarded in each item.

Supplementary Table 2 Quality assessment of included studies based on the Quality In Prognosis Studies (QUIPS).

| **Study** | **Quality evaluation of prognosis study** | | | | | | **Level of**  **Evidence^a^** |
| --- | --- | --- | --- | --- | --- | --- | --- |
|  | **Study**  **Participation** | **Study**  **Attrition** | **Prognostic**  **Factor**  **Measurement** | **Outcome**  **Measurement** | **Study Confounding** | **Statistical Analysis and**  **Reporting** |  |
| Duan et al^a^ | Yes | Partly | Yes | Yes | Partly | Yes | **2b** |
| Ma et al[20] | Yes | Partly | Partly | Partly | Partly | Partly | **2b** |
| Zhao et al[21] | Yes | Partly | Partly | Partly | Partly | Partly | **1b** |
| Ye et al[22] | Yes | Partly | Yes | Yes | Partly | Yes | **2b** |
| Feng et al[8] | Yes | Partly | Partly | Partly | Partly | Partly | **2b** |

^a^ The levels of evidence were estimated for all included studies with the Oxford Centre for Evidence Based Medicine criteria.


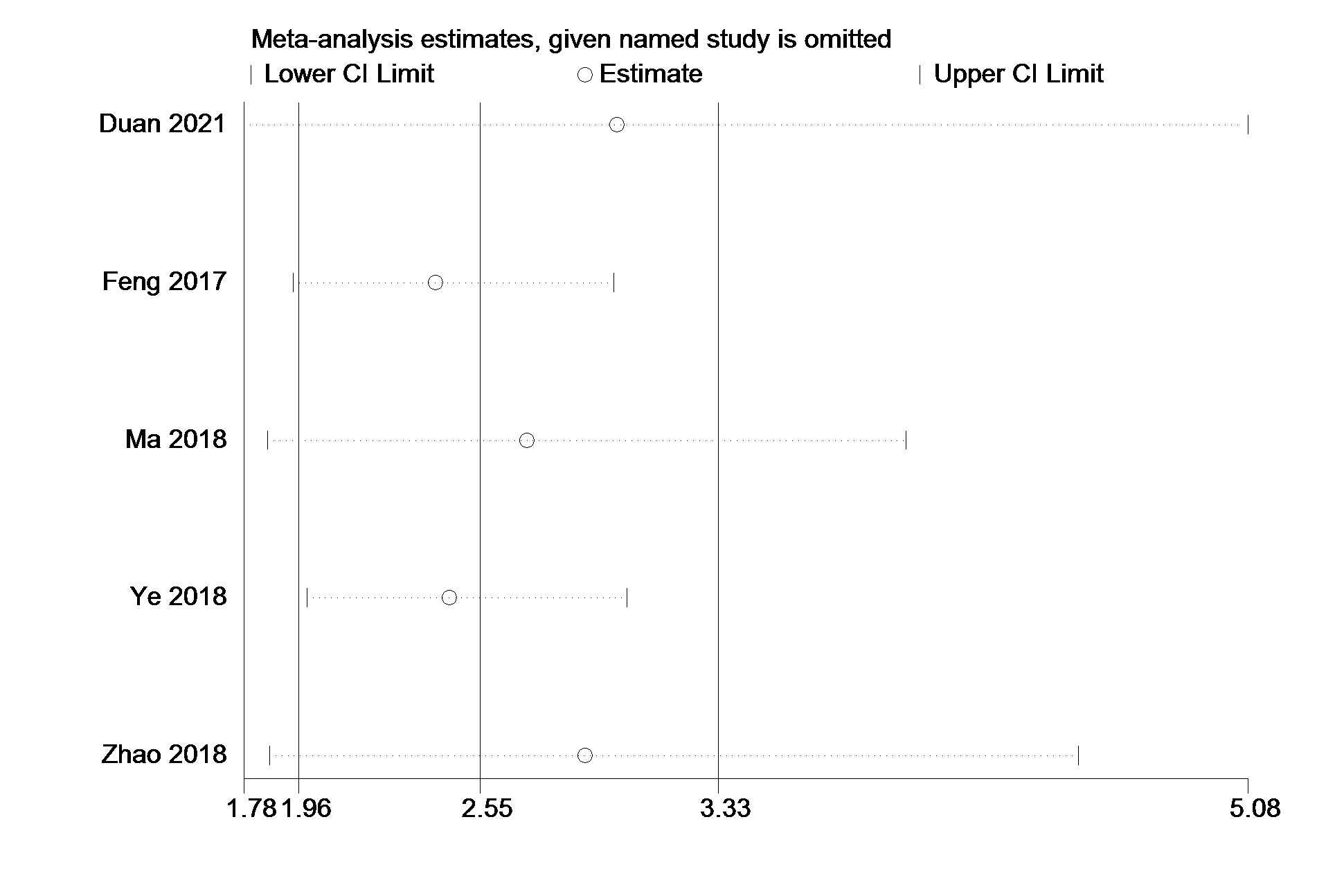


Supplementary Figure 1 Sensitivity analysis for OS of AFAP1-AS1 expression.


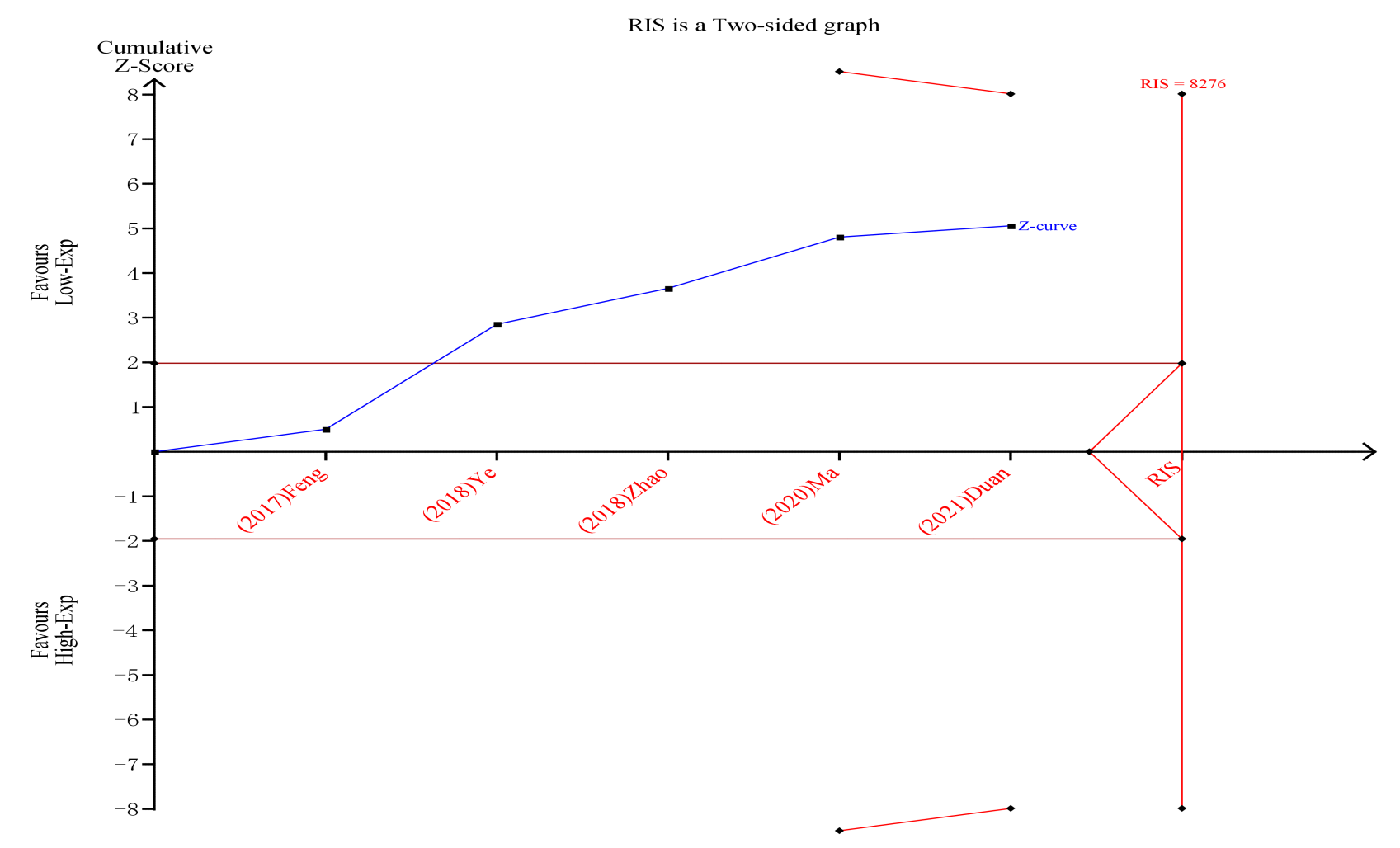


Supplementary Figure 2 Adjusted boundaries of trial sequence analysis.
